# Trends, correlates, and disease patterns of sedative-hypnotic use among elderly persons in Taiwan

**DOI:** 10.1186/s12888-022-03964-6

**Published:** 2022-05-04

**Authors:** Chia-Lun Kuo, I-Chia Chien, Ching-Heng Lin

**Affiliations:** 1 Tsaotun Psychiatric Center, Ministry of Health and Welfare, Nantou, Taiwan; 2 Department of Public Health, College of Medicine, National Cheng Kung University, Tainan, Taiwan; 3 Bali Psychiatric Center, Ministry of Health and Welfare, No. 33, HuaFuShan Rd, Bali District, New Taipei City, 249 Taiwan; 4 National Yang-Ming University, Taipei, Taiwan; 5 Taichung Veterans General Hospital, Taichung, Taiwan; 6 National Taipei University of Nursing and Health Sciences, Taipei, Taiwan

**Keywords:** Trends, Sedative-hypnotic, National Health Insurance, Taiwan, Psychotropic drug

## Abstract

**Objective:**

The population-based National Health Insurance database was adopted to investigate the prevalence, correlates, and disease patterns of sedative-hypnotic use in elderly persons in Taiwan.

**Methods:**

The National Health Research Institutes provided a database of 1,000,000 random subjects in the National Health Insurance program. We adopted this sample of subjects who were older than 65 years from 1997 to 2005 and examined the proportions of psychiatric and nonpsychiatric disorders with regard to sedative-hypnotic use.

**Results:**

The 1-year prevalence of sedative-hypnotic use in elderly individuals increased from 1.7% in 1997 to 5.5% in 2005. The 1-year prevalence rates of benzodiazepine (BZD) and non-BZD hypnotics were 3.2 and 3.1%, respectively, in 2005. The overall hypnotic use was highest in ≥85-year-olds, males, those with lower amounts of insurance or higher Charlson Comorbidity Index scores and those living in eastern Taiwan. Both BZD and non-BZD hypnotic use were most commonly used in nonpsychiatric disorders instead of psychiatric disorders. Among the psychiatric disorders, the disorders that accounted for higher BZD and non-BZD hypnotic use were senile and presenile organic psychotic conditions (3.4 and 3.4%, respectively). Higher BZD and non-BZD use was for diseases of the respiratory system (30.4 and 17.8%, respectively), the circulatory system (20.4 and 22.4%, respectively), and neoplasms (12.6 and 13.8%, respectively).

**Conclusion:**

The prevalence rates of both BZD and non-BZD sedative-hypnotic use increased from 1997 to 2005 in the elderly. The risk factors for sedative-hypnotic use were aging, male sex, lower insurance amount, and higher Charlson Comorbidity Index scores. Most BZD and non-BZD sedative-hypnotic users were persons with nonpsychiatric disorders.

## Introduction

Due to their relative safety compared with barbiturates, benzodiazepines (BZDs) are widely prescribed for many conditions, such as anxiety, insomnia, muscle relaxation, muscle spasticity, convulsive disorders, presurgical sedation, and detoxification from alcohol or other substances [1]. BZDs are divided into two categories on the basis of the Anatomical Therapeutic Chemical Classification System [2]), N05B Anxiolytics, which are mainly used for anxiety treatment, and N05C hypnotics and sedatives, which are for the treatment of insomnia and to induce sedation. In addition, nonbenzodiazepine drugs (Z-drugs), such as zopiclone, zolpidem and zoleplon, have also been prescribed for insomnia in all age groups commonly in recent decades due to their claims of safety and less or no disruption of sleep architecture [3]. The use of hypnotics has increased in recent decades, as has the prevalence of insomnia in many countries [4–6]. Among most studies, age was reported as one of the factors associated with increased sleep problems and hypnotic use [7]. A population-based survey in Beijing showed that the prevalence of insomnia in elderly individuals aged ≥65 years was 14.0%, while it was 9.2% in the all-age group, of which one-third reported taking BZDs as sleep-enhancing drugs [8]. In a community dwelling in urban Taiwan, 6% of elderly individuals aged ≧65 had insomnia over the past month, while the frequent use of hypnotics was 8.4% [9]. The prevalence of hypnotic use in the elderly varied greatly in different studies. In a study among Medicare home health recipients, 19% were prescribed benzodiazepines, and almost 7% were prescribed nonbenzodiazepine hypnotics [10]. Another population-based cross-sectional study in Brazil indicated that the prevalence of sleeping pill use was 14.3% in the age ≥ 60 group [11]. A study of six cohorts showed that the number might be higher in institutions, in which the prevalence of hypnotic use ranged from 14.5 to 22.9% [5].

Some correlates of hypnotic use in the elderly were reported. Female gender and advancing age were related to a higher prevalence of hypnotic use [12]. Regarding other demographic factors, marital status, such as being widowed, divorced or single, and having a low income or education level, were reported to be related to a higher prevalence of hypnotic use. Conversely, Z-drug use was seen more often in those with high education and high gross income [13]. Furthermore, a history of psychiatric or physical diseases and concomitant psychotropic or other CNS medications were also related to a higher prevalence of hypnotic use [14].

Although the proportion of elderly people using hypnotics has increased in recent decades, appropriateness has been widely discussed. One study demonstrated that inappropriate use was found for 100% of hypnotic BZD users and 65% of Z-drug users [15]. Additionally, an increased risk of adverse events such as falls, fracture, dizziness, and daytime sedation was common among elderly hypnotic users [16]. On the other hand, the relationship between hypnotic use and an increased risk of mortality or cognitive impairment is controversial [17]. Moreover, the prescription of hypnotics under heterogeneous underlying diseases is not yet well known.

Taiwan implemented the National Health Insurance program in March 1995, which offers comprehensive and universal health insurance to all citizens. The National Health Insurance database represents both the contemporary prescription and medical utilization patterns in Taiwan. In this study, we investigated the trends of sedative-hypnotic use within elderly persons as well as the factors associated with it. Additionally, the proportions of sedative-hypnotic use in the elderly for psychiatric and nonpsychiatric disorders were examined.

## Methods

The National Health Insurance database of medical claims in Taiwan includes outpatient care, inpatient care, and prescription drugs. The National Health Research Institutes provided a database of 1,000,000 random subjects in 2005, approximately 4.5% of the total population (22.6 million), to perform a related health services study. All the registration and claim data of these 1,000,000 individuals collected by the National Health Insurance program constitute the Longitudinal Health Insurance Database 2005. There were no statistically significant differences in age, sex, or average insured payroll-related amount between the sample group and all enrollees [18, 19]..

To analyze sedative-hypnotic use among elderly persons, we conducted a population-based, random sample study using data from 1997 to 2005. Persons older than 65 years on July 1 of each year with any record of sedative-hypnotic use were included. The initial sample consisted of 49,906 subjects in 1997, while in 1998 through 2005, 55,096, 60,193, 65,715, 71,387, 77,240, 83,460, 90,013 and 96,851 objects in each year were analyzed [18, 19]. We also identified the prevalence, correlates, and disease patterns of sedative-hypnotic use among the study subjects in 2005. This study was approved by the Tsaotun Psychiatric Center Institutional Review Board. Informed consent was not available from all participants in that we used claims data (a database established by the National Health Research Institute) in this study. Informed consent is waived by the Tsaotun Psychiatric Center Institutional Review Board**.** We confirm that all methods were carried out in accordance with relevant guidelines and regulations in this study.

### Assessment of Sedative-Hypnotic Use

In the current study, hypnotics and sedatives were recorded on the basis of the Anatomical Therapeutic Chemical Classification System (WHO, 2005), in which we divided hypnotics into 2 categories: BZD hypnotics (N05CD) and non-BZD hypnotics, which are also called Z-drugs (N05CF).

### Measures

Demographic data, including age, sex, insurance amount, geographic distribution, and urbanization, from the National Health Insurance database were analyzed. Age was stratified into three categories: 65–74; 75–84; and 85 years or older. In NHI, the premiums of the most insured are determined on the basis of the insured wage and premium rate. Insured wage is the insurance amount. The insured amount of the insured is divided into 38 grades ranging from NT$15,840 to NT$87,600. For the insured group without salaried income, the average premium remained at NT$1007. The insurance amount was classified into one of five categories: fixed premium, dependent, lower than US$640 (NTD 20,000), US$640–$1280 (NTD 20,000–39,999), and US$1281 (NTD 40,000) or more. Urbanization was divided into three categories: urban, suburban, and rural. The Charlson Comorbidity Index is widely used to predict future mortality for patients with a range of comorbid conditions. The CCI is a weighted index that takes into account the number and seriousness of comorbid disease [20]. In this study, the CCI was analyzed to determine the general health conditions of subjects.

### Assessment of Psychiatric Disorder

Subjects with hypnotics and sedative use who received at least one service claim in 2005 for either inpatient or outpatient care, with a primary or secondary diagnosis of a psychiatric disorder, were identified. We examined the proportion of psychiatric disorders with regard to sedative-hypnotic use. The psychiatric disorders were divided into major and minor psychiatric disorders. Major psychiatric disorders included International Classification of Diseases, Ninth Revision, clinical modification codes 290 through 299; minor psychiatric disorders included codes 300 through 316 [18, 19].

### Assessment of Medical Disorder

Subjects with sedative-hypnotic use who had at least one service claim in 2005 for either outpatient or inpatient care with the primary diagnosis of a medical disorder were also identified. The proportion of medical disorders recorded was examined with regard to sedative-hypnotic use [18, 19].

### Statistical Analysis

Considering the trends of sedative-hypnotic use in the elderly, we examined temporal changes from 1997 to 2005. Time series analysis was performed, and linear models were also used to assess the trends in sedative-hypnotic use. The adjustment factors used for multiple logistic analysis in Table 2, including age, sex, insurance amount, region, and urbanicity to analyze the significant factors associated with different groups of hypnotics. Data were fit into models using SAS for Windows, version 9.1, and the significance level was set at 0.05 [18, 19].

## Results

Table 1 shows the trends in sedative-hypnotic use in the elderly population from 1997 to 2005. The overall 1-year prevalence increased from 1.7 to 5.5% during that time period (*P* < 0.001). An increasing trend of sedative-hypnotics was noted in all three age groups: age 65 to 74, 75 to 84, and > =85. The hypnotics and sedative use increased from 1.6 to 4.2% in the 65–74 age group, 2.0 to 6.9% in the 75–84 age group, and 2.1 to 9.1% in the ≥85 age group (*p* < 0.001). The increase in hypnotics and sedative use was also noted in both sexes, from 1.6 to 6.0% in males (*p* < 0.001) and 1.9 to 4.9% in females (*p* < 0.001). The overall prevalence was higher in females than in males before 2003 and reversed after 2004. The increasing prevalence of hypnotics and sedatives from 1997 to 2005 was noted in both benzodiazepine derivative hypnotics (BZD hypnotic) and non-benzodiazepine-related hypnotics (non-BZD hypnotic), while the increasing trend was even more dominant in non-BZD hypnotics (*p* < 0.001). The prevalence of BZD hypnotic use increased from 1.5 to 3.2% (*P* = 0.013), while the prevalence of non-BZD hypnotic use increased from 0.2 to 3.1% (*P* < 0.001).

**Table 1:** Prevalence of sedative-hypnotic use from 1997 to 2005

	Year											
	1997	1998	1999	2000	2001	2002	2003	2004	2005	PD(%)	t ^a^	P
Sample	49, 906	55, 096	60, 193	65, 715	71, 387	77, 240	83, 460	90, 013	96, 51			
Age (y)												
65–74	1.6	1.8	2.1	2.1	2.4	2.8	2.9	3.3	4.2	163	9.01	< 0.001
75–84	2.0	2.5	2.8	2.9	3.4	4.0	4.3	5.5	6.9	245	8.17	< 0.001
≧85	2.1	1.5	3.1	3.5	3.6	4.8	5.1	6.9	9.1	333	7.57	< 0.001
Sex												
Male	1.6	1.9	2.2	2.3	2.7	3.3	3.3	4.5	6.0	275	6.72	< 0.001
Female	1.9	2.1	2.3	2.4	2.8	3.3	3.6	4.1	4.9	158	10.85	< 0.001
Sedative-Hypnotic												
BZD (N05CD)	1.5	1.6	1.7	1.6	1.7	1.8	1.8	2.2	3.2	113	3.30	0.013
Non-BZD (N05CF)	0.2	0.5	0.8	1.0	1.4	1.9	2.2	2.8	3.1	1450	23.05	< 0.001
Total	1.7	2.0	2.3	2.4	2.7	3.3	3.5	4.3	5.5	224	8.26	< 0.001

*PD* Percentage difference between 2005 and 1997

^a^Test for linear trend

Table 2 shows the logistic regression of factors associated with the prevalence of BZD sedative-hypnotic and non-BZD sedative-hypnotic use. With regard to age, both BZD and non-BZD sedative-hypnotic use was higher in the age > =85 group (*P* < 0.001). Moreover, the prevalence of BZD sedative-hypnotics (OR: 0.71, *P* < 0.001) was lower in females than in males, while non-BZD sedative-hypnotics showed no significant difference between sexes. There was no significant difference between different insurance amounts in the prevalence of BZD sedative-hypnotics. However, compared with the high insurance amount, higher non-BZD sedative-hypnotic use was higher in the fixed premium (OR: 2.30, *P* < 0.01), dependent groups (OR: 2.09, *P* < 0.01), and < 640 groups (OR: 1.90, *P* < 0.05). High correlations between the CCI and both BZD and non-BZD sedative-hypnotic use was also noted. The odds ratios in CCI ≥ 3 were more than 20 in the study (*P* < 0.001). However, lower BZD sedative-hypnotic use was found in urban areas (OR: 0.82, *P* < 0.001), while non-BZD hypnotic use was higher in suburban areas (OR: 1.12, *P* < 0.05). Regarding regions, both BZD and non-BZD sedative-hypnotic use rates were relatively lower in western Taiwan.

**Table 2:** Prevalence and logistic regression of sedative-hypnotic use

Variables	BZD (N05CD)			Non-BZD (N05CF)		
	Prevalence (%)	OR	95% CI	Prevalence (%)	OR	95% CI
Age						
65–74	2.4	1.00	–	2.6	1.00	–
75–84	4.1	1.52***	1.41–1.65	3.9	1.31***	1.21–1.42
≥ 85	6.2	2.56***	2.28–2.87	4.0	1.43***	1.25–1.63
Sex						
Male	3.8	1.00	–	3.3	1.00	–
Female	2.7	0.71***	0.65–0.76	3.0	0.96	0.89–1.04
Insurance amount ($)*						
Fixed premium	4.0	1.49	0.93–2.37	3.8	2.30**	1.32–4.01
Dependent	3.3	1.54	0.97–2.45	3.2	2.09**	1.20–3.65
< 640 (< 20,000 NTD)	2.8	1.20	0.75–1.91	2.8	1.90*	1.09–3.32
640–1280 (20,000–39,999 NTD)	2.8	1.42	0.82–2.45	2.4	1.71	0.91–3.23
≥ 1281(≥40,000 NTD)	2.0	1.00	–	1.4	1.00	–
Charlson Comorbidity Index						
0	0.5	1.00	–	0.5	1.00	–
1	2.6	5.47***	4.59–6.51	2.7	5.43***	4.57–6.46
2	6.2	12.82***	10.72–15.34	5.7	11.63***	9.73–13.90
3	10.4	23.08***	19.32–27.58	9.8	21.33***	17.89–25.45
Region						
Northern	3.2	0.59***	0.50–0.71	3.1	0.76**	0.63–0.93
Central	3.2	0.59***	0.49–0.71	3.7	0.93	0.76–1.14
Southern	3.2	0.59***	0.50–0.71	2.8	0.69***	0.57–0.84
Eastern	5.3	1.00	–	4.0	1.00	–
Urbanization						
Urban	3.2	0.82***	0.75–0.90	3.2	1.03	0.94–1.14
Suburban	3.3	0.97	0.87–1.08	3.4	1.12*	1.00–1.25
Rural	3.3	1.00	–	2.9	1.00	–
Total	3.2			3.1		

*: *p* < 0.05

**: *p* < 0.01

***: *p* < 0.001

Table 3 shows the proportions of psychiatric disorders among subjects according to BZD sedative-hypnotic and non-BZD sedative-hypnotic use. Psychiatric disorders were found in 8.60% of BZD sedative-hypnotic users and 10.87% of non-BZD sedative-hypnotic users. The proportions of major psychiatric disorders were 7.17% in BZD sedative-hypnotic users and 8.40% in non-BZD sedative-hypnotic users. Among the major psychiatric disorders, the disorders accounting for higher use of both BZD and non-BZD sedative-hypnotics were senile and presenile organic psychotic conditions (3.38 and 3.39%, respectively). Among the minor psychiatric disorders, neurotic disorders such as neurotic depression and anxiety state were associated with 1.46% in BZD sedative-hypnotic use and 2.21% in non-BZD sedative-hypnotic use, respectively.

**Table 3:** Psychiatric disorder proportions among subjects who used sedative-hypnotics

Medical Disorder (ICD-9-CM Code)	BZD (N05CD) (*n* = 3140)		Non-BZD (N05CF) (*n* = 3035)	
	n	%	n	%
**Without psychiatric disorder**	2870	91.40	2705	89.13
**Any psychiatric disorder**	270	8.60	330	10.87
**Any major psychiatric disorder**	225	7.17	255	8.40
Senile and presenile organic psychotic conditions (290)	106	3.38	103	3.39
Transient organic psychotic conditions (293)	10	0.32	14	0.46
Other organic psychotic conditions (294)	39	1.24	57	1.88
Schizophrenic disorders (295)	31	0.99	27	0.89
Affective psychoses (296)	42	1.34	63	2.08
Major depressive disorder (296.2, 296.3)	31	0.99	48	1.58
Bipolar affective disorder (296)	11	0.35	15	0.49
Paranoid states (297)	4	0.13	9	0.30
Other nonorganic psychosis (298)	5	0.16	5	0.16
**Any minor psychiatric disorder**	54	1.72	86	2.83
Neurotic disorders (300)	46	1.46	67	2.21
Anxiety state (300.0)	25	0.80	36	1.19
Anxiety disorders (300.0 except 300.01)	24	0.76	35	1.15
Neurotic depression (300.4)	16	0.51	27	0.89
Special symptoms or syndromes not elsewhere (307)	5	0.16	10	0.33
Depressive disorder, not elsewhere classified (311)	26	0.83	45	1.48

Table 4 shows the proportions of medical disorders among subjects according to BZD sedative-hypnotic and non-BZD sedative-hypnotic use. In nonpsychiatric disorders, there were higher BZD and non-BZD sedative-hypnotic use rates for diseases of the respiratory system, circulatory system, and digestive system.

**Table 4:** Proportions of medical disorders among the subjects using sedative-hypnotics

Medical Disorder (ICD-9-CM Code)	BZD (N05CD) (*n* = 2870)		Non-BZD (N05CF) (*n* = 2705)	
	n	%	n	%
**Without psychiatric disorder**				
Neoplasms (140-239)	361	12.58	374	13.83
Malignant neoplasm of trachea, bronchus and lung (162)	69	2.40	64	2.37
Malignant neoplasm of liver and intrahepatic bile ducts (155)	44	1.53	44	1.63
Diseases of the circulatory system (390-459)	586	20.42	606	22.40
Other forms of chronic ischemic heart disease (414)	89	3.10	140	5.18
Heart failure (428)	72	2.51	84	3.11
Occlusion of cerebral arteries (434)	111	3.87	107	3.96
Disease of the respiratory system (460-519)	873	30.42	482	17.82
Pneumonia, organism unspecified (486)	291	10.14	142	5.25
Chronic bronchitis (491)	103	3.59	121	4.47
Other diseases of lung (518)	352	12.26	79	2.92
Diseases of the digestive system (520-579)	287	10.00	283	10.46
Gastric ulcer (531)	54	1.88	61	2.26
Cholelithiasis (574)	48	1.67	35	1.29
Diseases of the genitourinary system (580-629)	189	6.59	233	8.61
Other disorders of urethra and urinary tract (599)	69	2.40	91	3.36
Diseases of the musculoskeletal system and connective tissue (710-739)	143	4.98	267	9.87
Osteoarthrosis and allied disorders (715)	49	1.71	84	3.11
Injury and poisoning (800-999)	250	8.71	294	10.87
Fracture of neck of femur (820)	58	2.02	75	2.77

## Discussion

In this study, the overall prevalence of sedative-hypnotic use increased from 1.7 to 5.5% from 1997 to 2005. The increased prevalence of sedative-hypnotics might be attributable to the comprehensive NHI program through which the evaluation and treatment of psychiatric and medical diseases has become available and affordable. However, the prevalence was lower than those in surveys in Brazil and Iceland [9, 21]. When considering the high prevalence of antipsychotic and antidepressant use in elderly individuals in Taiwan compared with other countries [19, 20], the lower prevalence of sedative-hypnotic use was not undertreatment of mental disorders in Taiwan. Another possible explanation is that in our study, sedative-hypnotic use was based on the Anatomical Therapeutic Chemical Classification System [2], in which we divided anxiolytics, hypnotics and sedatives into 2 categories: anxiolytic (N05B) and sedative-hypnotics (N05C). Some elderly individuals might take anxiolytics for insomnia but were not included in the hypnotic use group.

Increasing trends were noted in all three age groups: 65 to 74, 75 to 84, and > =85. This was consistent with other studies that revealed that the prevalence rates of insomnia and sedative-hypnotic use increased with age [4, 19]. Considering the higher prevalence of depression in the age group > = 85 in a previous community study of older people in Taiwan [22], insomnia as one symptom of depression could be more common among the elderly. In addition, an increase in BZD sedative-hypnotic use was noted in both sexes, while in females, it was higher than that in males from 1997 to 2003 but significantly reversed from 2004 to 2005. Considering the higher prevalence of depression and antidepressant use with time in females [20], whether males tend to take mere sedative-hypnotics for insomnia instead of treating potential psychiatric diseases requires further investigation.

The popularity of the National Health Insurance might lead to the consistency of treatment for psychiatric disorders and sedative-hypnotic use in different insurance amounts. A higher prevalence of sedative-hypnotics was highly related to low income, which was presented by the insurance amount in our study. This is reasonable since a higher prevalence of depression and insomnia in subjects with lower income was reported in some studies [23–25]. Moreover, a lower incidence of sedative-hypnotic use in western Taiwan and urban areas might suggest caution in clinicians’ prescription of sedative-hypnotic use in urban areas. This might also imply that the prevalence rates of depression and medical service utilization could vary when considering urbanization [26].

The Charlson Comorbidity Index is a weighted index that takes into account the number and seriousness of comorbid diseases [18]. In our study, a high correlation was noted between the CCI and both BZD and non-BZD sedative-hypnotic use. It is well known that chronic diseases increase as the risk of depression [27, 28], in which insomnia might be a core symptom. Some studies have even indicated that insomnia and sedative-hypnotic use had a stronger relation to somatic health than to mental health in the elderly group [29].

For sedative-hypnotic use in psychiatric disorders, only 8.60% of BZD sedative-hypnotic users and 10.87% of non-BZD sedative-hypnotic users had at least one psychiatric disorder. The prevalence of sedative-hypnotic use for the treatment of insomnia was close to the prevalence of insomnia in old age in Taiwan [9]. In the users with psychiatric disorders, the most common diagnosis was senile organic psychotic disorder. Hypnotic use in dementia is for the treatment of related altered sleep cycles or behavioral and psychological symptoms of dementia at night. BZD prescription in the elderly might be underestimated since many of them used anxiolytics for sleep problems due to the fewer side effects of daytime sedation.

Surprisingly, with respect to medical disorders in BZD sedative-hypnotic use, the highest proportion was in diseases of the respiratory system. Nearly one-third of BZD sedative-hypnotic users had a primary diagnosis of diseases of the respiratory system. Non-BZD sedative-hypnotic users had a lower but still significant proportion of diseases of the respiratory system. Patients with diseases of the respiratory system might suffer from poor sleep quality, and sedative-hypnotics might be prescribed [30]. However, some studies mentioned the relationship between the use of sedative-hypnotics, BZDs or non-BZDs, with an increased risk of pneumonia or other respiratory diseases [31, 32]. Additionally, respiratory depression due to sedative-hypnotic use was well known in previous studies [33]. There could be a bidirectional relationship between sedative-hypnotic use and respiratory diseases. Clinicians should employ more caution when prescribing both BZD and non-BZD sedative-hypnotics in this older and respiratory-vulnerable population.

Another common medical disorder in BZD and non-BZD sedative-hypnotic use was the circulatory system. The use of sedative-hypnotics may be due to insomnia as a symptom of the depression and anxiety found commonly in patients with circulatory conditions [34, 35]. However, studies on the risk of mortality from heart disease in sedative-hypnotic uses are inconsistent [36, 37]. Some studies even indicated that hypnotic use might be related to lower mortality [38]. Further studies about the safety of prescribing sedative-hypnotics in patients with circulatory conditions are required.

For BZDs and non-BZDs, sedative-hypnotic use in neoplasms, which was the third most common primary diagnosis in medical disorders, accounted for more than 10% of all sedative-hypnotic uses. Patients with cancer might suffer pain and poor sleep quality, leading to the use of sedatives and hypnotics. The risk of cancer in sedative-hypnotic use has been studied in recent years. Most studies indicated that sedative-hypnotic use was not associated with an overall increase in cancer risk when considering confounding factors such as smoking [39], while others indicated the opposite conclusion [40].

There are some limitations in our study. First, the disease pattern was estimated through the registration of diagnostic codes in the National Health Insurance system, and the records were not designed for research purposes. The quality of records may vary depending on different hospitals and clinicians. The subjects with psychiatric disorders might be underestimated due to missed coding by clinicians or overestimated due to the requirements of The National Health Research Institutes for the prescriptions of sedative-hypnotics. Second, the prescription patterns of sedative-hypnotics, such as dosage, duration, or frequency, were not investigated in this study. Third, the impairment of cognition and dementia in medical subjects could be missed, and therefore, the subjects with psychiatric disorders could be underestimated. Finally, other limitations include the cross-sectional nature of the study, the absence of a comparison group (e.g., those with psychiatric diagnoses listed who did not use hypnotics), and the fact that nonmedication therapies were not investigated or reported.

## Conclusion

The prevalence rates of both BZD and non-BZD use increased from 1997 to 2005 in the elderly population in Taiwan. The overall sedative-hypnotic use was highest in ≥85-year-olds, those with lower insurance amounts and higher Charlson Comorbidity Index scores and those in eastern Taiwan. Both BZDs and non-BZDs were most commonly used in nonpsychiatric disorders instead of psychiatric disorders. Further investigations of the indication, dosage, frequency, duration, adverse effects, and off-label uses of sedative-hypnotics in the elderly are required.

## Data Availability

A database established by the National Health Research Institute (NHRI), which included medical claim records of outpatient care, inpatient care and prescription drugs, was used. The datasets generated and/or analyzed during the current study are not publicly available due to ownership by NHRI but are available from the corresponding author on reasonable request.

## References

[1] Hollister LE, Muller-Oerlinghausen B, Rickels K, Shader RI (1993). Clinical uses of benzodiazepines. J Clin Psychopharmacol.

[2] WHO (2005). Guidelines for ATC Classification and DDD Assignment.

[3] Barbera J, Shapiro C (2005). Benefit-risk assessment of zaleplon in the treatment of insomnia. Drug Saf.

[4] Calem M, Bisla J, Begum A, Dewey M, Bebbington PE, Brugha T, Stewart R (2012). Increased prevalence of insomnia and changes in hypnotics use in England over 15 years: analysis of the 1993, 2000, and 2007 National Psychiatric Morbidity Surveys. Sleep.

[5] Ruths S, Sorensen PH, Kirkevold O, Husebo BS, Kruger K, Halvorsen KH, Selbaek G (2013). Trends in psychotropic drug prescribing in Norwegian nursing homes from 1997 to 2009: a comparison of six cohorts. Int J Geriatr Psychiatry.

[6] Machado-Alba JE, Alzate-Carvajal V, Jimenez-Canizales CE (2015). Trends in the consumption of anxiolytic and hypnotic drugs in a Colombian population. Rev Colomb Psiquiatr.

[7] Benard-Laribiere A, Noize P, Pambrun E, Bazin F, Verdoux H, Tournier M, Pariente A (2016). Comorbidities and concurrent medications increasing the risk of adverse drug reactions: prevalence in French benzodiazepine users. Eur J Clin Pharmacol.

[8] Xiang YT, Ma X, Cai ZJ, Li SR, Xiang YQ, Guo HL, Ungvari GS (2008). The prevalence of insomnia, its sociodemographic and clinical correlates, and treatment in rural and urban regions of Beijing, China: a general population-based survey. Sleep.

[9] Su TP, Huang SR, Chou P (2004). Prevalence and risk factors of insomnia in community-dwelling Chinese elderly: a Taiwanese urban area survey. Aust N Z J Psychiatry.

[10] Cotton BP, Lohman MC, Brooks JM, Whiteman KL, Bao Y, Greenberg RL, Bruce ML (2017). Prevalence of and Factors Related to Prescription Opioids, Benzodiazepines, and Hypnotics Among Medicare Home Health Recipients. Home Healthc Now.

[11] Kodaira K, Silva MT (2017). Sleeping pill use in Brazil: a population-based, cross-sectional study. BMJ Open.

[12] Doi Y, Minowa M, Okawa M, Uchiyama M (2000). Prevalence of sleep disturbance and hypnotic medication use in relation to sociodemographic factors in the general Japanese adult population. J Epidemiol.

[13] Andersen AB, Frydenberg M (2011). Long-term use of zopiclone, zolpidem and zaleplon among Danish elderly and the association with sociodemographic factors and use of other drugs. Pharmacoepidemiol Drug Saf.

[14] Asplund R (2000). Sleep and hypnotic use in relation to perceived somatic and mental health among the elderly. Arch Gerontol Geriatr.

[15] Neutel CI, Skurtveit S, Berg C (2012). What is the point of guidelines? Benzodiazepine and z-hypnotic use by an elderly population. Sleep Med.

[16] Glass J, Lanctot KL, Herrmann N, Sproule BA, Busto UE (2005). Sedative hypnotics in older people with insomnia: meta-analysis of risks and benefits. BMJ.

[17] Billioti de Gage S, Moride Y, Ducruet T, Kurth T, Verdoux H, Tournier M, Begaud B (2014). Benzodiazepine use and risk of Alzheimer’s disease: case-control study. BMJ.

[18] Kuo CL, Chien IC, Lin CH, Cheng SW (2015). Trends, correlates, and disease patterns of antidepressant use among elderly persons in Taiwan. Soc Psychiatry Psychiatr Epidemiol.

[19] Kuo CL, Chien IC, Lin CH (2016). Trends, correlates, and disease patterns of antipsychotic use among elderly persons in Taiwan. Asia Pac Psychiatry.

[20] Charlson ME, Pompei P, Ales KL, MacKenzie CR (1987). A new method of classifying prognostic comorbidity in longitudinal studies: development and validation. J Chronic Dis.

[21] Linnet K, Gudmundsson LS, Birgisdottir FG, Sigurdsson EL, Johannsson M, Tomasdottir MO, Sigurdsson JA (2016). Multimorbidity and use of hypnotic and anxiolytic drugs: cross-sectional and follow-up study in primary healthcare in Iceland. BMC Fam Pract.

[22] Chong MY, Tsang HY, Chen CS, Tang TC, Chen CC, Yeh TL, Lo HY (2001). Community study of depression in old age in Taiwan: prevalence, life events and socio-demographic correlates. Br J Psychiatry.

[23] Koster A, Bosma H, Kempen GI, Penninx BW, Beekman AT, Deeg DJ, van Eijk JT (2006). Socioeconomic differences in incident depression in older adults: the role of psychosocial factors, physical health status, and behavioral factors. J Psychosom Res.

[24] Deyessa N, Berhane Y, Alem A, Hogberg U, Kullgren G (2008). Depression among women in rural Ethiopia as related to socioeconomic factors: a community-based study on women in reproductive age groups. Scand J Public Health.

[25] da Cunha RV, Bastos GA, Del Duca GF (2012). Prevalence of depression and associated factors in a low income community of Porto Alegre, Rio Grande do Sul. Rev Bras Epidemiol.

[26] Strothers HS, Rust G, Minor P, Fresh E, Druss B, Satcher D (2005). Disparities in antidepressant treatment in Medicaid elderly diagnosed with depression. J Am Geriatr Soc.

[27] Mojtabai R, Olfson M (2004). Major depression in community-dwelling middle-aged and older adults: prevalence and 2- and 4-year follow-up symptoms. Psychol Med.

[28] Moussavi S, Chatterji S, Verdes E, Tandon A, Patel V, Ustun B (2007). Depression, chronic diseases, and decrements in health: results from the World Health Surveys. Lancet.

[29] Asplund R (2000). Sleep and hypnotic use in relation to perceived somatic and mental health among the elderly. Arch Gerontol Geriatr.

[30] Vozoris NT, Fischer HD, Wang X, Anderson GM, Bell CM, Gershon AS, Rochon PA (2013). Benzodiazepine use among older adults with chronic obstructive pulmonary disease: a population-based cohort study. Drugs Aging.

[31] Jung S, Spence MM, Escasa NM, Lee EA, Hui RL, Gibbs NE (2016). The Risk of Pneumonia in Older Adults Using Nonbenzodiazepine Hypnotics. J Manag Care Spec Pharm.

[32] Taipale H, Tolppanen AM, Koponen M, Tanskanen A, Lavikainen P, Sund R, Hartikainen S (2017). Risk of pneumonia associated with incident benzodiazepine use among community-dwelling adults with Alzheimer disease. CMAJ.

[33] Chung WS, Lai CY, Lin CL, Kao CH (2015). Adverse respiratory events associated with hypnotics use in patients of chronic obstructive pulmonary disease: a population-based case-control study. Medicine (Baltimore).

[34] Ahto M, Isoaho R, Puolijoki H, Laippala P, Romo M, Kivela SL (1997). Coronary heart disease and depression in the elderly--a population-based study. Fam Pract.

[35] Mendes de Leon CF, Krumholz HM, Seeman TS, Vaccarino V, Williams CS, Kasl SV, Berkman LF (1998). Depression and risk of coronary heart disease in elderly men and women: New Haven EPESE, 1982-1991. Established populations for the epidemiologic studies of the elderly. Arch Intern Med.

[36] Kim YH, Kim HB, Kim DH, Kim JY, Shin HY (2018). Use of hypnotics and the risk of or mortality from heart disease: a meta-analysis of observational studies. Korean J Intern Med.

[37] Mesrine S, Gusto G, Clavel-Chapelon F, Boutron-Ruault MC, Fournier A (2018). Use of benzodiazepines and cardiovascular mortality in a cohort of women aged over 50 years. Eur J Clin Pharmacol.

[38] Lan TY, Zeng YF, Tang GJ, Kao HC, Chiu HJ, Lan TH, Ho HF (2015). The use of hypnotics and mortality--a population-based retrospective cohort study. PLoS One.

[39] Pottegard A, Friis S, Andersen M, Hallas J (2013). Use of benzodiazepines or benzodiazepine related drugs and the risk of cancer: a population-based case-control study. Br J Clin Pharmacol.

[40] Sivertsen B, Salo P, Pentti J, Kivimaki M, Vahtera J (2015). Use of sleep medications and risk of cancer: a matched case-control study. Sleep Med.

