# Correction: Trends, correlates, and disease patterns of sedative-hypnotic use among elderly persons in Taiwan

**DOI:** 10.1186/s12888-022-04004-z

**Published:** 2022-06-01

**Authors:** Chia-Lun Kuo, I-Chia Chien, Ching-Heng Lin

**Affiliations:** 1 Tsaotun Psychiatric Center, Ministry of Health and Welfare, Nantou, Taiwan; 2 Department of Public Health, College of Medicine, National Cheng Kung University, Tainan, Taiwan; 3 Bali Psychiatric Center, Ministry of Health and Wel- fare, No. 33, HuaFuShan Rd, Bali District, New Taipei City, 249 Taiwan; 4 National Yang-Ming University, Taipei, Taiwan; 5 Taichung Veterans General Hospital, Taichung, Taiwan; 6 National Taipei University of Nursing and Health Sciences, Taipei, Taiwan

**Correction: BMC Psychiatry 22, 316 (2022)**

**https://doi.org/10.1186/s12888-022-03964-6**

Following the publication of the original article [1], the authors identified that the funding note was incorrect. The correct funding note is given below.
